# A green TLC–densitometric bioanalytical method for concurrent determination of duloxetine and risperidone in human plasma with in-silico DDI evaluation

**DOI:** 10.1186/s13065-025-01715-8

**Published:** 2026-01-21

**Authors:** Fatma Magdy, Raghda A. Emam, Basma H. Anwar

**Affiliations:** 1 Medicinal Chemistry Department, Faculty of Pharmacy, Beni-Suef University, Alshaheed Shehata Ahmad Hegazy St, Beni-Suef, 62514 Egypt; 2 Pharmaceutical Analytical Chemistry Department, Faculty of Pharmacy, Beni-Suef University, Alshaheed Shehata Ahmad Hegazy St, Beni- Suef, 62514 Egypt

**Keywords:** Green chemistry, Insilico DDI prediction, Duloxetine, Spiked human plasma, Risperidone, Thin layer chromatography

## Abstract

Schizophrenia is a chronic, complex and severs psychiatric disorder that impacts men and women globally. The combined use of atypical antipsychotics; risperidone (RSP), and antidepressants medications; duloxetine hydrochloride (DLX), has grown more clinical significance in cutting edge treatments of complex psychiatric conditions like schizophrenia. This research targets to develop an environmentally harmonious, selective, and accurate TLC method to measure the levels of both DLX and RSP in spiked human plasma samples. A mixture of methanol, ethylacetate, and 33% ammonia solution (6: 4: 0.2, by volume) was successfully utilized as a solvent system to separate the proposed components. A UV-scanning wavelength of 230 nm was used for detection. An internal standard; propranolol (PRP); was used to account for small variations in the samples. The resulting retardation factors (R_f_) were 0.02, 0.32, 0.46 and 0.59 for plasma, DLX, PRP and RSP, respectively. The linearity ranges were 0.04–0.4 and 0.1–0.6 µg band^− 1^ for DLX and RSP, respectively. The suggested method’s environmental safety was evaluated using five tools and the results showed good greenness and blueness method’s characteristics. Additionally, the method’s figures of merits were within the accepted criteria, according to the US-FDA bio-analytical guidelines. Furthermore, drug-drug interactions (DDI) evaluation was conducted using web-based DDI tool to examine the interaction manifestations and to verify safety and therapeutic effectiveness of this combination.

**Graphical abstract:**

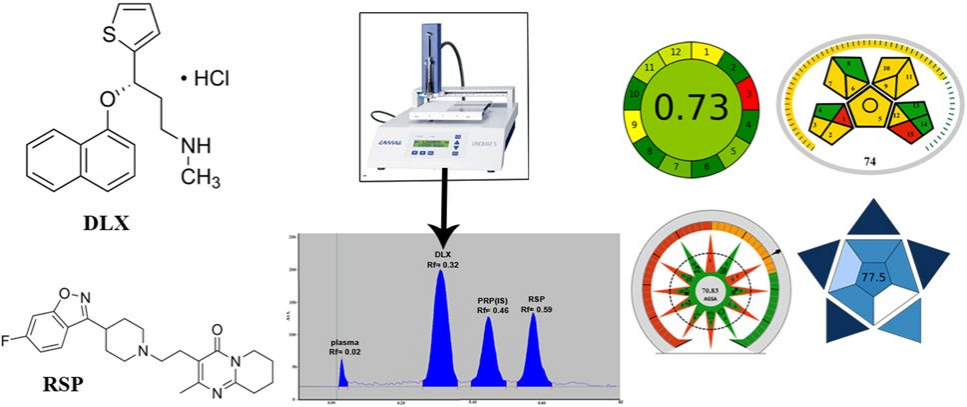

## Introduction

Schizophrenia; a devastating psychiatric disorder; affects 1% of people worldwide, striking men and women equally. The disease is defined by two types of symptoms: positive and negative or manic-depressive mood. The negative symptoms include paranoid delusions, hallucinations, loss of normal abilities, social isolation and lack of motivation [1]. Tragically, the struggle with schizophrenia is so profound that there is a 10% lifetime risk of suicide for those affected [1].

**Fig. 1:**
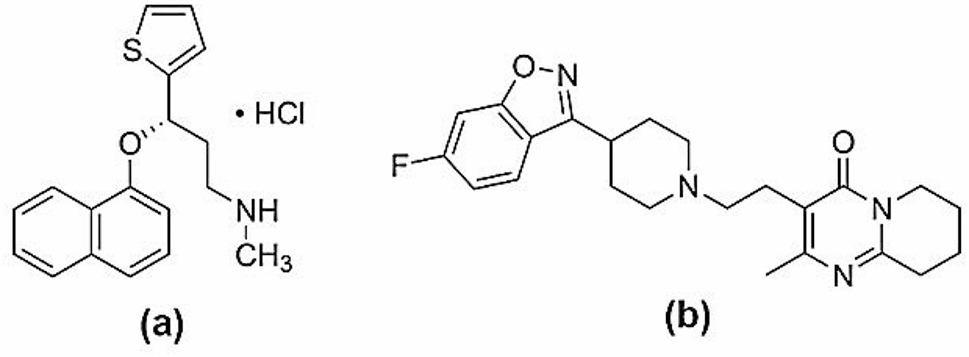
The chemical structures of (a) duloxetine hydrochloride and (b) risperidone

Duloxetine hydrochloride (DLX); as illustrated in Fig. 1; is a type of medications family called a serotonin-norepinephrine reuptake inhibitors [2]. It’s primarily used to manage conditions like anxiety and depression, and it also helps to alleviate nerve pain related to diabetic peripheral neuropathy. Patients taking DLX may also experience improved mood, better sleep, enhanced appetite, and higher energy, along with reduced nervousness [2]. Scientists have developed a variety of ways to measure DLX levels in different matrices, such as: TLC [3, 4], HPLC [5–7], voltammetry [8], UV-spectrophotometry [9, 10] and spectrofluorimetry [11–13].

Risperidone (RSP) (Fig. 1) belongs to the benzisoxazole class of antipsychotic medications [2]. This novel therapeutic agent shows remarkably strong binding to serotonin receptors type, 5-HT_2_, considerable receptors binding capacity for dopamine D_2_, and notable strong binding for both α_1_- and α_2_-adrenergic, as well as histamine H_1_ receptors [14]. Clinical applications include managing of schizophrenia and related psychotic disorders, along with short-duration therapy for acute manic or mixed episodes in bipolar disorder patients. Within the United States, pediatric use of RSP extends to treating irritability symptoms in autism spectrum disorders [2]. Previous research has employed diverse analytical approaches to determine RSP concentrations, either as a single analyte or combined with impurities and co-administered medications across different sample types. These methodologies have included HPLC [15–17], TLC [18–20], spectrophotometric techniques [21, 22], and voltammetric analyses [23, 24].

Currently, thin layer chromatography (TLC) represents a highly effective instrumental approach and extensively utilized analytical methodology for examining pharmaceutical compounds [25–27], food products [28–30], plant materials [31, 32], environmental specimens [33, 34], and biological matrices [35–39]. Although HPLC/UPLC offers superior resolution, accuracy and precision over TLC method, TLC represents an optimal balance of analytical performance, practicality, and accessibility for several benefits, including the capacity to quantify multiple samples concurrently while consuming minimal solvent volumes, potential for automation, straightforward sample preparation protocols, reduced experimental duration and costs, along with rapid modification of chromatographic parameters, detection capabilities, and concurrent processing of multiple chromatographic separations [40].

The bioanalysis field encompasses the quantitative determination of compounds, pharmaceuticals and their metabolic products within biological fluids, tissues or matrices, including urine, serum, blood, plasma, and tissue homogenates. Currently, this field play an important role in the pharmacokinetic and pharmacodynamic evaluation of new medications throughout their lifecycle; from initial discovery through successive phases of pharmaceutical development until its regulatory approval in the markets [41].

Green analytical chemistry emphasizes the development of analytical methodologies that minimize environmental impact and enhance human safety. The environmental sustainability of analytical procedures is evaluated through multiple parameters, including reagent quantity and toxicity, waste production, operational complexity, size reduction, energy consumption and automation capabilities [42]. Various assessment tools have been used to assess the ecological influence of analytical methods. Those evaluation systems involve the national environmental method index (NEMI) [43], analytical eco-scale [44], green analytical procedure index (GAPI) [45], the modified green analytical procedure index (MoGAPI) [46], the analytical greenness (AGREE) metric [42], the analytical green star area (AGSA) [47], and environmental, performance, and practicality index (EPPI) [48] which have gained widespread adoption due to their practical applicability and compatibility with diverse analytical techniques [35, 36, 49–51]. Each evaluation tool presents distinct strengths and limitations; consequently, certain metrics have gained preference among researchers as they offer more comprehensive quantitative assessments of methodological environmental performance [46].

The combination of DLX with RSP appears to represent a well-tolerated and effective therapeutic approach for addressing primary negative symptoms in schizophrenia, demonstrating particular value for patients who have achieved clinical stability on antipsychotic medications but continue to experience inadequate symptom improvement, particularly regarding negative manifestations [52]. Current literature reveals an absence of chromatographic methodologies specifically developed for the simultaneous analysis of these two compounds. Consequently, this investigation presents a US-FDA validated thin-layer chromatographic approach for sensitive and quantitative determination and separation of DLX and RSP in laboratory-synthesized mixtures and human plasma specimens augmented with these analytes, demonstrating superior selectivity and precision. Moreover, a thorough evaluation of potential drug-drug interactions (DDI) was conducted using validated web-based DDI assessment tools to examine the severity of potential interaction manifestations and to verify both the safety and therapeutic effectiveness of this therapeutic regimen. Additionally, the ecological sustainability of the proposed methodology was assessed through five distinct evaluation frameworks: modified GAPI (MoGAPI) [46], AGREE [42], analytical green star area (AGSA) [47], environmental, performance, and practicality index (EPPI) [48], and blue applicability grade index (BAGI) [53].

## Experimental

### Instruments and equipment

- Thin-layer chromatography plates (20 × 20 cm) made of aluminum and coated with silica gel 60 F254 stationary phase having 0.25 mm thick layer and particles measuring 5 μm in diameter (Merck, Germany).
- Samples were applied using a Camag Linomat IV automatic application system fitted with a 100 µL syringe.
- Densitometric analysis was conducted employing a TLC scanner Camag (Muttenz, Switzerland) (model 3 S/N) operated through version 3.15 software of winCATS.
- Plasma protein precipitation and separation were achieved using an electric centrifuge operating at low speed of 4000 rpm (Zjmzym, China).

### Material and reagents

#### Pure standards

- Eva Pharm Company (Giza, Egypt) supplied DLX with a certified purity of 99.91%.
- RSP was obtained, with certificate analysis indicating a purity of 99.10%, from Sigma Aldrich Egypt,
- Company for Pharmaceuticals; Al-Kahira (Giza, Egypt), provided PRP, which possessed a certified purity level of 99.21%.
- Blank plasma specimens were generously supplied by El-Mokhtabar Laboratory (Beni-suef, Egypt). This research received ethical approval of the Faculty of Pharmacy, Beni-Suef University Research Ethics Committee under approval number REC-H-PhBSU-24,022.

#### Chemicals and reagents

- Methanol of HPLC purity grade was purchased from Fischer, UK.
- Ethyl acetate and 33% ammonia solution were obtained from EL-Nasr Pharmaceutical Chemical Company, in Cairo, Egypt.

### Standard solutions

Individual stock solutions containing 1 mg mL⁻¹ of DLX, RSP and PRP were prepared individually using methanol as the dissolution medium.

###  Blank plasma sample

Human plasma (1 mL) was precisely transferred to a 10-mL measuring flask and diluted to volume with methanol solvent. Plasma proteins were then precipitated, followed by centrifugation and subsequently, the supernatant was gathered for analysis.

## Procedures

### Chromatographic conditions

Sample application was performed using a Camag Linomat IV applicator, depositing 10 µL volumes as 3 mm-wide bands positioned 10 mm above the plate baseline, with inter-band spacing of 6 mm. The mobile phase system comprising methanol: ethyl acetate: ammonia (6: 4: 0.2, by volume) was introduced into the development chamber and equilibrated for 15 min to achieve vapor saturation. TLC plates were positioned with their lower edges in contact with the solvent system, sealed appropriately, and allowed to develop completely. Subsequently, chromatographic peaks were visualized at 230 nm using UV densitometry.

### Calibration curves construction and QC samples analysis

#### Pure samples preparation

Aliquots representative to 0.04–0.50 mg of DLX and 0.08–0.6 mg for RSP were quantitatively mounted from their pure standard solutions (1 mg mL^− 1^) into two 10-mL volumetric flasks distinct series. Subsequently, 0.5 mL of IS (PRP) stock solution (1 mg mL^− 1^) was added to each flask, and the final volumes were set to the graduation mark using methanol. Analytical determinations were performed using triplicate 10 µL injections for each sample, adhering to the procedures outlined in (Sect. 3.1). Calibration curves for each proposed compound were established by graphing the integrated peak area ratios (analyte of interest peak area relative to that of the IS) varied with concentrations (µg band^− 1^).

*Spiked human plasma samples Preparation* Aliquots equivalent to 0.04–0.40 mg of DLX and 0.10–0.60 mg for RSP were separately and quantitatively pipetted from their corresponding standard solutions (1 mg mL^− 1^) into two different series of 10-mL volumetric flasks. Each flask was supplemented with 1 mL of human plasma in addition to 0.5 mL of IS (PRP) solution, followed by vortex mixing for 1 min prior to volume adjustment to the graduation mark with methanol. Plasma protein precipitation was achieved through centrifugation at specified conditions for 5 min. Triplicate 10 µL aliquots of the resulting filtrate were spotted to TLC plates, and the analytical procedures detailed in (Sect. 3.1) were executed. Calibration curves for the plasma-spiked samples were assembled by plotting integrated peak area ratios (analyte of interest peak area relative to that of the IS) versus drug concentrations (µg band^− 1^). In accordance with FDA guidelines, quality control (QC) samples encompassing the lower limit of quantitation (LLOQ) in addition to low, middle and high QC; LQC, MQC and HQC, respectively [54], were formulated and assayed under identical conditions for each cited drug.

## Results and discussion

Planar chromatographic techniques encompass diverse applications, from basic screening procedures to sophisticated instrumental quantitative determinations across various sample types and matrices. Thin-layer chromatography (TLC) represents one category of planar chromatographic approaches that offers economic and temporal efficiency benefits [39, 40, 55]. This methodology demonstrates broad utility in pharmaceutical analysis [25, 26], characterization of impurities and degradation compounds [27, 56], and isolation plus separation of biomedical metabolites or components from various biological fluids requiring minimal sample preparation [35–39]. Additionally, it facilitates the resolution of structurally similar compounds within complex mixtures [40, 55].

Consequently, this investigation presents an environmentally assessed TLC methodology for concurrent assay of DLX and RSP in binary mixtures and augmented human plasma specimens. The developed approach provides a TLC protocol characterized by simplicity, environmental sustainability, and cost-effectiveness, while enabling both identification and quantification of these two pharmaceutical agents in plasma matrices containing the proposed added analytes.

### In-silico assessment of drug-drug interaction (DDI) severity

**Fig. 2:**
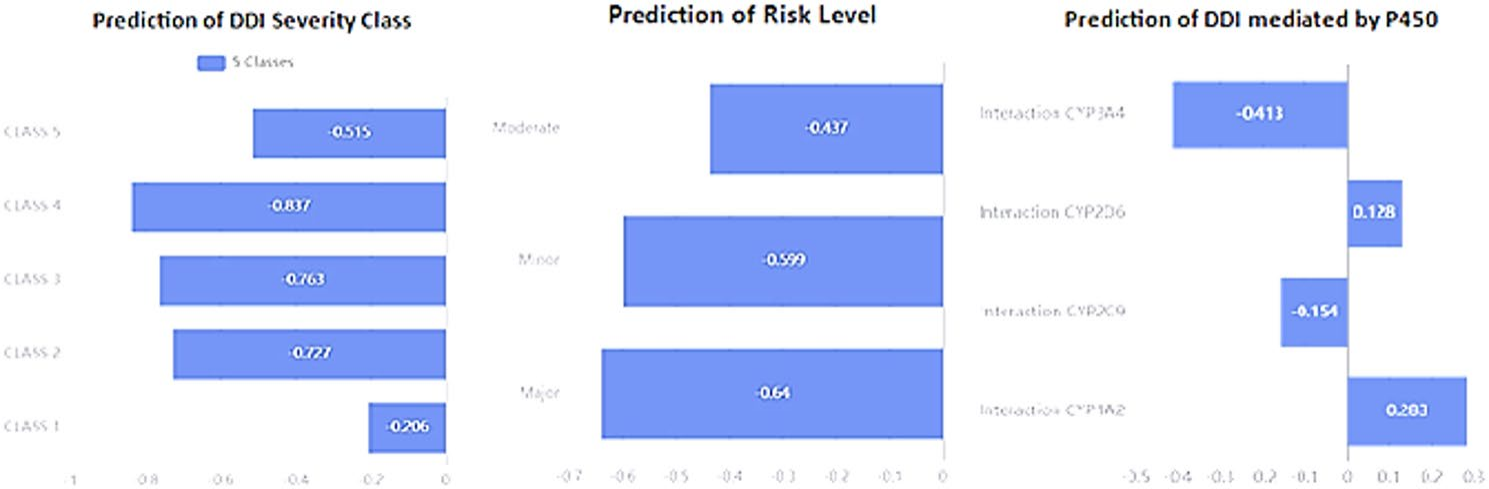
Graph showing DDI severity class and IAP values at five CYP450 enzymes level for DLX- RSP DDI using *Way2drug* online prediction tool

When patients administered multiple medications at the same time, a risk of clinically meaningful DDIs often exists, that makes it important to thoroughly understand the systems of cytochrome P450 enzyme to assure patient safety [57]. The main liver enzymes that involved in drug metabolism are CYP3A4, CYP2C9, CYP2D6, and CYP1A2, hence they are responsible for breaking down of roughly 80% of drugs that undergo CYP1, 2, 3- oxidative metabolism [58]. To predict potential CYP450-related molecular structure interactions between our cited drugs; DLX and RSP, Way*2*drug computational platform was used [59]. The Invariant Accuracy of Prediction (IAP) approach was applied, which computed the interaction likelihood by comparing the change between the probability of active (p.a.) and inactive (Pi) interactions for each cytochrome enzyme. Drugs interact with CYP-enzyme system in three main ways: they can be metabolized as substrates, block enzyme function (as inhibitors) or speed up enzyme activity (metabolic enhancers), affecting other co-prescribed drug bio-processing. When acting as inhibitors, those drugs can increase bloodstream levels of another co-prescribed drug or levels of those drugs themselves leading to potential toxic effects. Whereas, upon enzyme induction or enhancement, plasma levels of drugs metabolized by those same pathways may decrease, leading to decreased therapeutic effects. The insilico analysis consistently showed low IAP values for all four CYP450 enzymes examined: CYP1A2 (0.283), CYP2C9 (-0.154), CYP2D6 (0.128), and CYP3A4 (-0.413), for DLX and RIS combined administration, as shown in Fig. 2. Further safety assessment evaluated adverse effects from co- administration drugs, with most active probability (Pa) values staying lower than their corresponding inactive (Pi) thresholds. Given that significant adverse effects require Pa to exceed Pi, those results showed that, for all enzymes pathways, Pi was greater than Pa, except for tachycardia DDI, indicating low likelihood of interaction as demonstrated in Table 1, that indicated low adverse effects from prescribing DLX and RIS together. These results were supported by the ORCA severity classification system that assigned them 5 classes negative designation i.e. no measured interactions, Fig. 2. Interpretation of IAP follows standard guidelines that positive values more than 0.7 show significant interaction potential, while positive values lower than 0.7, indicate lower interaction potential that of low clinical evidence, and negative results suggest low interaction likelihood; as our study showed. The leave-one-out cross-validation (LOOCV) method confirmed these elucidations using complement software for predicting the biological activity profile, named PASS training datasets, which compared experimental data for all drug with structure-activity relationships (SAR) resulting in low risk level predicted. Those in-silico findings beside the developed analytical method results show the possibility of safe co-administration of DLX along with RIS without compromising drug effectiveness. Although computational modeling offers useful direction for prioritizing drug development and studying combination therapy, it remains a supplementary tool that cannot replace conventional in vivo- and in vitro- laboratory validation methods.

**Table 1:** Probable adverse effects that results from concurrent administration of DLX with RSP

Activity	Pa^*^	Pi^*^
DDI arrhythmia	0.013	0.685
DDI bradicardia	0.044	0.477
DDI hypertension	0.023	0.411
DDI hypotension	0.029	0.578
DDI qt_interval_prolongation	0.005	0.811
DDI tachycardia	0.158	0.093

^*^ Pa is the active interaction probability and Pi is the inactive interaction probability at individual cytochrome P450 enzyme levels

### Method optimization

To achieve optimal separation and detection performance, multiple variables were examined, including:

#### The mobile system

**Fig. 3:**
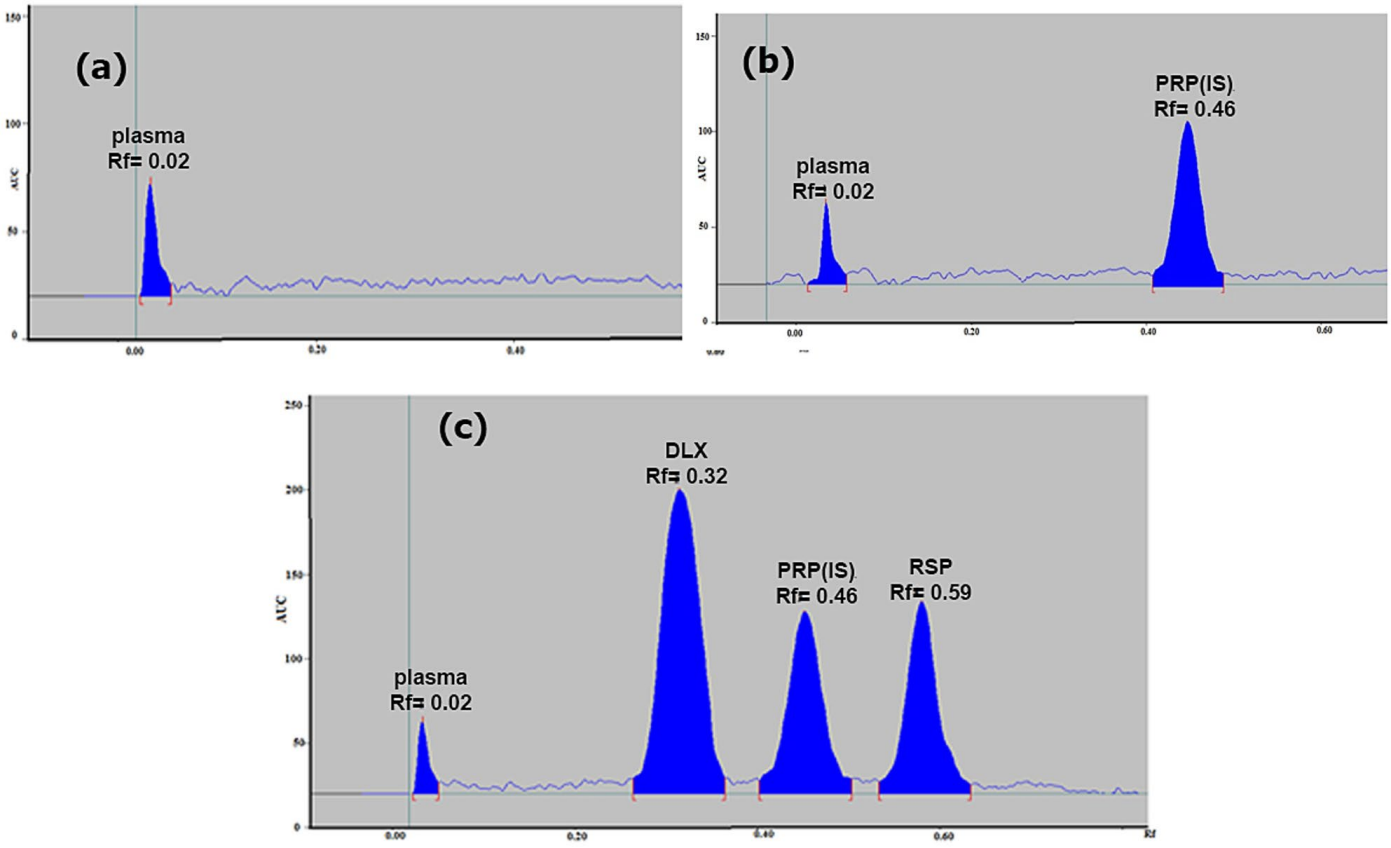
2D chromatogram of (a) blank human plasma, (b) plasma and 0.5 µg band^− 1^ of the internal standard propranolol, (c) spiked human plasma with 0.2 µg band^− 1^ of duloxetine hydrochloride (R_f_ = 0.32), 0.5 µg band^− 1^ of the internal standard propranolol (R_f_ = 0.46), and 0.3 µg band^− 1^ risperidone (R_f_ = 0.52) and, using methanol: ethyl acetate: ammonia (6: 4: 0.2, by volume) as a developing system at 230 nm

Multiple developing solvent systems underwent evaluation to achieve optimal separation of the three analytes designated as DLX, RSP, and the plasma peak, with the objective of identifying the most appropriate conditions for resolution and selectivity. Initial investigations employed combinations of environmentally sustainable solvents, specifically ethanol, methanol, acetone and ethyl acetate. The preliminary assessment commenced with binary mixtures of acetone and ethanol at volumetric ratios of (4: 6, v/v) and (3: 7, v/v), respectively, in which DLX and RSP were well resoluted, RSP peak was sharp and symmetric, but DLX peak was tailed, broad and attached to the baseline and plasma peak. So, ethanol were replaced with a less green solvent; methanol; and a system of methanol/ acetone with ratio of (6: 4, v/v) was tried, but DLX peak was still tailed and attached to the baseline. Other trials were performed by replacing acetone with ethylacetate and using methanol/ ethylacetate mixture in ratio (6: 4, v/v), where DLX peak moved far from base line and was well-separated from plasma and RSP peaks, but DLX peak still tailed and broad. Accordingly, small ratios of 33% ammonia solution was added to the mobile phase in ratios of 0.05, 0.1, 0.15 and 0.2, by volume, to improve DLX peak shape, where 0.2 mL was the optimum one. Ultimately, the investigation culminated in the selection of a ternary mobile phase composition of methanol: ethyl acetate: ammonia at volume ratios of (6: 4: 0.2, by volume), respectively, as depicted in Fig. 3.

#### UV densitometric wavelength

Optimal detection sensitivity for the target compounds was attained at a scanning wavelength of 230 nm (Fig. 3), following systematic evaluation of multiple wavelengths including 230, 245, 254, 280, and 300 nm.

#### Internal standard selection

A comprehensive screening of potential internal standards was conducted, encompassing paracetamol, propranolol, nortriptyline, dapoxetine hydrochloride and tadalafil. Among the evaluated compounds, propranolol (PRP) displayed optimal characteristics for internal standard application.

###  Method development

Quantitative calibration relationships for the pharmaceutical compounds under investigation were established by graphical representation of peak area ratios (analyte signal relative to internal standard signal) against pharmaceutical concentrations (µg band^− 1^). Linear regression analyses were performed within the working ranges of 0.04–0.40 µg band^− 1^ for DLX and 0.10–0.60 µg band^− 1^ for RSP. Statistical parameters and acceptance standards are presented in Table 2. The chromatographic system achieved baseline separation with retardation factor (R_f_) values of 00.02, 0.32, 0.46 and 0.59 for plasma, DLX, PRP and RSP, respectively, as illustrated in Fig. 3c.

**Table 2:** Assay and method validation parameters for the determination of Duloxetine hydrochloride and Risperidone by the proposed TLC method

Parameters	Pure		Spiked human plasma	
	DLX	RSP	DLX	RSP
Calibration range (µg band^− 1^)	0.04–0.50 (4–50 µg mL^− 1^)	0.08–0.60 (8–60 µg mL^− 1^)	0.04–0.40 (4–40 µg mL^− 1^)	0.10–0.60 (10–60 µg mL^− 1^)
Slope	0.4548	0.1832	0.3361	1.3751
Intercept	0.0149	0.0076	0.0122	0.0905
Correlation coefficient	0.9997	0.9998	0.9997	0.9997
Accuracy	99.64	99.56	99.55	100.14
Robustness parameters (RSD%) ^a^ - Methanol (6 ± 0.1 mL) - Ethyl acetate (4 ± 0.1 mL) - Saturation time (15 ± 5 min) - Ammonia (0.2 ± 0.01 mL)			1.75	1.68
			1.99	1.89
			1.61	1.53
			1.72	1.90
LLOQ (µg band^− 1^)			0.04	0.10
ULOQ (µg band^− 1^)			0.40	0.60

^a^ the %RSD was calculated for the R_f values_

### Method validation

Regarding FDA validation protocols [54], there is widespread consensus that quantitative bioanalytical methodologies should incorporate assessment of key validation criteria: calibration model, accuracy (encompassing bias and precision), selectivity, and stability.

#### Analytical calibration and quantification limits

Linear response relationships were created for plasma specimens augmented with the targeted drugs within the concentration intervals of 0.04–0.4 µg band^− 1^ for DLX and 0.1–0.6 µg band^− 1^ for RSP. The LLOQ and ULOQ levels were ascertained to be 0.04 and 0.4 µg band^− 1^ for DLX and 0.1 and 0.6 µg band^− 1^ for RSP, respectively, as depicted in Table 2.

#### Intra- and inter-day precision and accuracy assessment

Four distinct QC specimens; LLOQ, LQC, MQC, and HQC; were validated to substantiate the Intra- and inter-day precision and accuracy of the developed methodology. The designated QC concentration levels were (0.04, 0.1, 0.2, and 0.3) µg band^− 1^ for DLX, and (0.1, 0.2, 0.3, and 0.5) µg band^− 1^ for RSP. Drug concentrations in the test specimens were calculated through substitution into the regression equations referenced in Table 2, with findings tabulated in Table 3.

**Table 3:** Intra and inter assay precision and accuracy of LLOQ, LQC, MQC and HQC of Duloxetine hydrochloride and Risperidone in spiked human plasma samples

Component	Concentration (µg band^− 1^) ^a^		Intra-day			Inter-day		
			Recovery %	RSD %	Bias % ^b^	Recovery %	RSD %	Bias % ^b^
DLX	**LLOQ**	**0.04**	85.44	1.55	-14.56	92.94	2.56	-7.06
	**LQC**	**0.1**	92.93	1.67	-7.07	88.75	3.11	-11.25
	**MQC**	**0.2**	98.87	2.05	-1.13	85.09	3.32	-14.91
	**HQC**	**0.3**	93.78	4.84	-6.22	87.26	2.59	-12.74
RSP	**LLOQ**	**0.1**	95.26	2.46	-4.74	98.87	1.34	-1.13
	**LQC**	**0.2**	102.32	1.99	2.32	112.56	5.07	12.56
	**MQC**	**0.3**	105.77	2.89	5.77	114.30	5.89	14.30
	**HQC**	**0.5**	109.89	3.68	9.89	110.88	4.70	10.88

^a^ Average of 3 experiments

^b^ Bias = [(measured concentration - nominal concentration)/nominal concentration] x 100

#### Chromatographic selectivity assessment

The densitometric chromatographic profiles presented in Fig. 3 confirmed the analytical selectivity and chromatographic discrimination capability of the established methodology through exemplary baseline resolution achieved between the target analytes plasma, DLX, RSP, and internal standard PRR.

#### Stability assessment

The chemical stability and degradation resistance of the drugs under investigation was validated through two stability protocols, including three freeze-thaw cycles and bench-top stability evaluations. The detailed stability results were documented in Table 4.

**Table 4:** Stability results of Duloxetine hydrochloride and Risperidone in spiked human plasma at different conditions using the proposed TLC method

The analyte	Recovery %^a^		
	Concentration (µg band^− 1^)	Three freeze thaw cycles ^b^	Bench top stability
DLX	0.1	96.19	83.22
	0.2	93.42	85.75
	0.3	90.33	89.32
Mean ± % RSD		93.31 ± 2.57	86.09 ± 2.91
RSP	0.2	87.56	77.78
	0.3	95.04	80.45
	0.5	92.11	91.15
Mean ± % RSD		91.57 ± 3.36	83.13 ± 6.95

^a^ Average of 3 determinations

^b^ Freezing was done at -20 °C

#### System suitability criteria

The overall analytical approach’s operational performance was validated through calculation of critical chromatographic measurements, encompassing: resolution, peak symmetry, and selectivity factors (α). Experimental results were evaluated against internationally recognized acceptance specifications [60], as presented in Table 5.

**Table 5:** Parameters of system suitability of the developed TLC method for the determination of Duloxetine hydrochloride and Risperidone

Parameters	Plasma	DLX		PRP		RSP	Reference value [60]
Capacity factor (K’)	-	2.13		1.17		0.69	0–1
Symmetry factor	-	0.92		0.92		0.96	~ 1
Resolution (Rs)	5.44		1.61		1.55		*R* > 1.5
Selectivity (α)	15.18		1.82		1.70		α > 1

#### The extraction efficiency

Analytical extraction performance was quantified and statistically evaluated through comparative assessment of integrated peak responses between samples subjected to extraction procedures and corresponding unprocessed reference standard ones. The extraction process was validated and the recovery results were presented in Table 6.

**Table 6:** The extraction recovery results of Duloxetine hydrochloride and Risperidone in spiked human plasma

DLX		RSP	
Concentration (µg band^− 1^)	**% Recovery** ^**a**^	**Concentration (µg band** ^**− 1**^ **)**	**% Recovery** ^**a**^
0.1	94.42	0.2	89.14
0.2	91.00	0.3	85.74
0.3	96.91	0.5	90.06
Mean ± % RSD	94.11 ± 2.57	88.31 ± 2.10	

^a^ Average of 3 determinations

#### Robustness

The analytical method’s robustness was investigated through intentional minor variations across operational variables, including mobile phase compositional ratios, spectrophotometric detection wavelengths, and jar saturation time. Comprehensive robustness assessment results are demonstrated in Table 2.

### Greenness and blueness assessment of the developed TLC method

The field of green analytical chemistry (GAC) represents a fundamental paradigm for advancing environmental sustainability within analytical and quality control laboratory environments [61]. Drawing from green chemistry theory, the twelve fundamental GAC principles establish a structured methodology for creating environmentally conscious analytical approaches [62]. The twelve GAC principles encompass: direct analytical approaches, small sample volume, in-situ analysis, integration of analytical procedure, automation and miniaturization, avoidance of derivatization reactions, waste minimization with waste treatment systems, high-throughput capabilities, energy-efficient consumption patterns, implementation of renewable reagent alternatives, toxicity reduction, and enhanced operator safety protocols [63]. Contemporary approaches typically involve the adoption of environmentally benign solvents or complete solvent elimination, optimization of reagent utilization, reduction of energy requirements, avoidance of derivatization processes, and mitigation of waste production [64–66]. The development and implementation of green assessment metrics represents a critical methodology for evaluating the comparative sustainability of conventional and emerging analytical procedures [67, 68]. Multiple assessment metrics are presently employed to quantify the ecological impact of analytical methodologies. The ecological performance of the proposed approach was conducted through four distinct evaluation frameworks: the modified green analytical procedure index (MoGAPI) [46], the analytical greenness metric approach (AGREE) [42], the analytical green star area (AGSA) [47], and the blue applicability grade index (BAGI) [53].

####  Modified green analytical procedure index (MoGAPI)

The MoGAPI evaluation tool facilitates the provision of a comprehensive visual assessment of methodological safety and ecological impact parameters, while generating a cumulative score for each analytical approach. This framework delivers a graphical representation that elucidates individual steps of the analytical workflow. Furthermore, it enhances comparative evaluations between methodologies based on their aggregate scores, particularly when procedural variations are substantial. Additionally, the software streamlines and expedites MoGAPI implementation. The computational software utilized for score calculation and MoGAPI assessment generation is publicly accessible online source at bit.ly/MoGAPI [46]. Moreover, MoGAPI enables method classification into three categories: excellent green (≥ 75), acceptable green (50–74), and inadequately green (< 50). The overall score in the MoGAPI evaluation is displayed on the chart, and the chromatic scaling surrounding the pentagrams signifies the comprehensive method evaluation, which is determined through summation of all individual scores and subsequent division by the maximum achievable value [46].

**Fig. 4:**
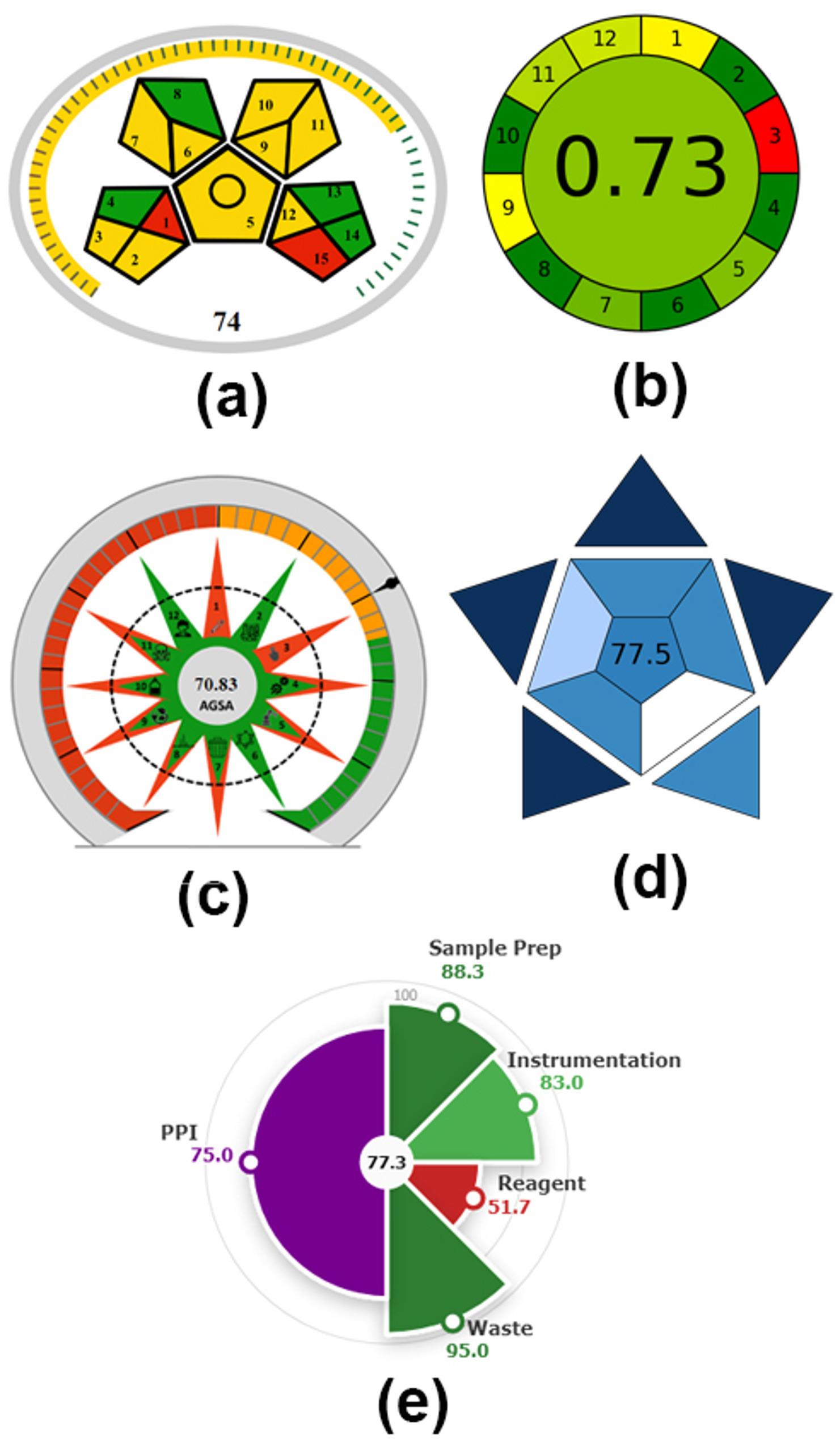
Greenness and blueness assessment of the developed TLC method, **(a)** MoGAPI figure, **(b)** AGREE figure, **(c)** AGSA figure, **(d)** EPPI figure, and **(e)** BAGI figure

With respect to the developed methodology, samples were obtained through offline collection procedures, requiring physical preservation protocols (samples were maintained under frozen conditions) and transportation logistics, followed by micro-extraction processes without further treatment procedures. Environmentally sustainable solvents, including methanol-ethylacetate-33% ammonia solution, were employed in volumes spanning 10 to 100 mL. The instrumental energy requirements were ≤ 1.5 kWh per specimen, incorporating hermetic sealing protocols and generating 1–10 mL waste per sample. The methodology is applied for quantitative analysis of DLX and RSP compounds. As demonstrated in Fig. 4a, the cumulative score is 74, which categorizes the established method as possessing intermediate environmental sustainability characteristics [46].

#### Analytical greenness metric approach (AGREE)

The analytical AGREEnness calculator constitutes a comprehensive, versatile, and user-friendly assessment framework that generates readily interpretable and informative analytical outputs. The evaluation metrics systematically reflect the twelve foundational principles underlying GAC and are normalized to a standardized 0–1 scale, whereby the final metric is computed based on these established criteria. The analytical output comprises a chromatic circular pictogram demonstrating the overall score, methodological performance across individual assessment parameters, identification of weakness and strength domains, and researcher-specified weighting factors. Open-access software facilitates the assessment methodology. The platform is publicly available as open-source software and may be obtained from https://mostwiedzy.pl/AGREE [42].

Concerning the developed TLC methodology, the analytical process employs external sample preparation with minimal steps’ number, and requires 10 µL of plasma specimen. Analysis was performed offline. The methodology is semi-automated and incorporates miniaturization. Derivatization reagents are eliminated from the analytical process. Three analytes (DLX, RSP and PRP) are detected in one analytical run. LC represents the highest energy-consuming analytical method. The mobile phase components (methanol and ethylacetate) are classified as flammable, while ammonia is categorized as corrosive. AGREE evaluation produced a score of 0.73 for the TLC methodology (Fig. 4b). The generated pictogram showed a green coloration in its central region, confirming the adequate environmental sustainability of the proposed method [42].

#### Analytical green star area (AGSA)

The AGSA evaluation tool delivers a thorough and systematic assessment of analytical method sustainability by methodically quantifying their correspondence with the twelve fundamental principles of GAC. The associated software is accessible as open-source technology at bit.ly/AGSA2025, enabling interdisciplinary comparative analyses [47]. As illustrated in Fig. 4c, the scoring framework is engineered to assess multiple principles of analytical procedures. Each principle is evaluated through targeted inquiries with three-level response options, permitting progressive differentiation among methodologies. Enhanced scores reflect superior sustainability, prioritizing small sample manipulation, reduced energy utilization, non-toxic reagents, and waste reduction approaches. These numerical values are cumulative, aggregated to achieve a maximum of 36 points (12 principles × 3 points), constituting 100% completion [47].

The developed methodology necessitated extensive sample preparation procedures and utilized size below 0.1 g of sample. Samples were transported to remote laboratory. Certain procedural elements were integrated, thereby reducing operational steps and minimizing instrumental requirements. The proposed approach was semi-automated, and derivatization procedures were not required. The waste volume generated was maintained below 100 mL per sample, with no waste disposal protocols reported. Additionally, a combination of renewable and non-renewable reagents was employed, exhibiting toxicity levels ranging from 3 to 5 pictograms. Three target analytes were simultaneously analyzed per analytical run, utilizing low-risk procedures that required minimal personal protective equipment, and the energy consumption of the analytical process ranged between 1 and 1.5 kW per sample. The established method demonstrates excellent environmental sustainability according to the AGSA metric with a score of 70.83 [47], as presented in Fig. 4c.

#### Environmental, performance, and practicality index (EPPI)

The EPPI framework comprises two complementary indices: the environmental impact (EI) Index and the performance and practicality index (PPI). These innovative, broadly applicable, and accessible metrics enable holistic assessment of analytical methodologies through concurrent consideration of environmental sustainability, analytical efficacy, and practical feasibility. The EI Index incorporates GAC principles alongside green sample preparation (GSP) guidelines to evaluate environmental compatibility throughout the entire analytical workflow, encompassing pre-synthesis procedures, sample preparation protocols, and measurement techniques. Conversely, the PPI addresses analytical performance (redness) and practical applicability (blueness). EPPI outcomes are presented in dual format: quantitative scores ranging from 1 to 100 and qualitative pictograms depicted as pie charts, wherein green sectors denote environmental sustainability while purple sectors represent the integrated contributions of performance (redness) and practicality (blueness), as illustrated in Fig. 4d. The EPPI too is available online at this link https://reemobaydo.github.io/EI-PPI-Project/ [48].

The left half represents the EI score; the higher EI score the greener the method. The score of (85–100) denotes an ideal green method (dark green), the score of (85 − 70) represents an environmentally friendly method (light green), the score of (70 − 55) indicates moderate environmental impact (yellow), and the score of (< 55) reflects the high environmental impact (red). In the other hand, the right half represents the PPI score, the higher PPI score the more practical the method. The score of (75–100) represents the excellent practicality (dark purple), The score of (50–74) represents the accepted practicality (light purple), and the score of (< 50) represents the impractical method (pink) [48]. The EPPI overall score of the proposed method was calculated and found to be 77.3; with EI score of 79.5 (light green) and PPI score of 75 (dark purple), as shown in Fig. 4d, indicating that the proposed method is environmentally friendly method with excellent practicality [48].

#### Blue applicability grade index (BAGI)

BAGI serves as a supplementary evaluation methodology to existing green evaluation frameworks (e.g., AGREE, MoGAPI, AGSA). It focuses on the “blue” tenets of white analytical chemistry that primarily address practical implementation aspects. BAGI incorporates ten evaluation parameters to produce both a visual pictogram and numerical score that characterizes the applicability and operational efficacy of analytical methodologies. A progressive blue chromatic spectrum was implemented to represent the final assessment, employing varying gradations of dark blue, blue, light blue, and white to denote high, medium, low, and zero adherence to the specified parameters, respectively. The numerical value displayed in the central region of the resulting figure denotes a comprehensive rating assigned to the analytical technique, spanning a range of 25 to 100. The poorest methodological efficacy regarding applicability corresponds to a score of 25, whereas a score of 100 signifies exceptional method efficacy. To achieve “practical” classification, it is recommended that methodologies attain a minimum threshold of 60 points. The practical applicability assessment of analytical methods is conducted through either an application (mostwiedzy.pl/bagi) or an equivalent online platform (bagi-index.anvil.app) [53].

Within the proposed TLC methodology, separation and determination of the proposed medications were accomplished using UV spectrophotometric detection, yielding quantitative analytical results. The analytical procedure facilitated the detection of three analytes (DLX, RSP and PRP). Concerning instrumentation requirements, simple equipment (TLC-UV) commonly available in most laboratory settings was utilized. Simple and low-cost sample preparation procedures were performed (protein precipitation and filtration) with sample throughput exceeding 10 samples per hour. Pre-concentration step was not required. Semi-automated operations using conventional instrumentation were performed. The sample size was 10 µL. Consequently, the overall score of 77.5 was achieved for the established methodology, indicating its favorable applicability characteristics [53], as illustrated in Fig. 4e.

## Conclusion

An environmentally conscious TLC methodology was estimated, optimized and validated for the quantitative determination of DLX and RSP in binary mixtures and spiked human plasma specimens, utilizing PRP as the internal standard. The developed approach demonstrated high sensitivity, operational simplicity, rapid analysis time, cost-effectiveness, environmental compatibility, and reduced ecological impact. All method validation parameters satisfied FDA regulatory requirements. Assessment using five distinct environmental evaluation metrics confirmed that the proposed TLC procedure exhibits excellent green analytical characteristics and environmental sustainability.

## Data Availability

The datasets used and/or analyzed during the current study are available from the corresponding author upon reasonable request.
